# Identification and validation of novel DNA methylation markers for early diagnosis of lung adenocarcinoma

**DOI:** 10.1002/1878-0261.12767

**Published:** 2020-08-27

**Authors:** Miao Li, Chen Zhang, Lijun Zhou, Siyu Li, Yuan Jie Cao, Longlong Wang, Rong Xiang, Yi Shi, Yongjun Piao

**Affiliations:** ^1^ School of Medicine Nankai University Tianjin China; ^2^ Department of Radiation and Oncology National Clinical Research Center for Cancer and Tianjin Key Laboratory of Cancer Prevention and Therapy Tianjin Medical University Cancer Institute and Hospital Tianjin China; ^3^ Tianjin Key Laboratory of Human Development and Reproductive Regulation Nankai University Affiliated Hospital of Obstetrics and Gynecology Tianjin China

**Keywords:** DNA methylation, early diagnosis, feature selection, lung adenocarcinoma

## Abstract

Lung cancer has the highest mortality of all cancers worldwide. Epigenetic alterations have emerged as potential biomarkers for early diagnosis of various cancer tissue types. To identify methylation markers for early diagnosis of lung adenocarcinoma, we aimed to integrate genome‐wide DNA methylation and gene expression data from The Cancer Genome Atlas. To this end, we first examined the global DNA methylation pattern of lung adenocarcinoma and investigated the relationship between DNA methylation subtypes and clinical features. We then extracted differentially methylated and expressed genes, and adopted feature selection techniques to determine the final methylation markers. The performance of the markers in predicting lung adenocarcinoma was evaluated on three independent datasets from Gene Expression Omnibus. Protein levels of marker genes were validated by immunohistochemistry, and their biological function was further verified *in vivo*. We identified three novel methylation markers in lung adenocarcinoma including cg08032924, cg14823851, and cg19161124, mapping to *CMTM2*, *TBX4*, and *DPP6*, respectively. Validating these results on three independent datasets indicated that the three markers can achieve extremely high sensitivity and specificity in distinguishing lung adenocarcinoma from normal samples. Immunohistochemistry quantification results confirmed that markers are weakly expressed in human lung adenocarcinoma, and *CMTM2* decreased tumor growth of mouse Lewis lung carcinoma *in vivo*. Overall, our study identified three novel methylation markers in lung adenocarcinoma which may contribute toward an improved diagnosis potentially leading to a better outcome for patients with lung adenocarcinoma.

## Graphical abstract

Lung cancer has the highest mortality of all cancers worldwide. To identify epigenetic markers for early diagnosis of lung adenocarcinoma (LUAD), we integrated genome‐wide DNA methylation and gene expression analyses. We identified methylation signatures in three genes (*CMTM2*, *TBX4,* and *DPP6*) for early diagnosis of LUAD. Our findings may contribute toward an earlier LUAD diagnosis, potentially improving outcomes for patients.

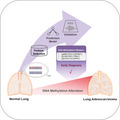

**Abbreviations**
1st exon: the first exon
AUC: area under the curve
BMP: bone morphogenetic protein
CGI: CpG islands
CIMP: CpG island methylation phenotype
DEG: differentially expressed gene
DMC: differentially methylated cytosine
GEO: gene expression omnibus
GR: gain ratio
IG: information gain
IHC: immunohistochemistry
LUAD: lung adenocarcinoma
NSCLC: non‐small‐cell lung cancer
RF: reliefF
ROC: receiver operating characteristic
SU: symmetrical uncertainty
TCGA: the cancer genome atlas

## 1 Introduction

Lung cancer is the leading cause of death with cancer worldwide [1]. An estimated 72 000 deaths in men and an estimated 63 220 deaths in women from lung cancer occurred in the United States alone in 2020 [2]. As the major histological type, non‐small‐cell lung cancer (NSCLC) accounts for ~ 80% of all lung cancer cases, in which lung adenocarcinoma (LUAD), arising from the mucus‐secreting glandular cells, accounts for ~ 50% [3]. The average 5‐year survival rate in the United States for the patients diagnosed with lung cancer during 2009 through 2015 was as low as 19%. It is noticeable that the survival rates varied most among different stages of lung cancer. The 5‐year relative survival rate is only 5% for patients diagnosed with metastatic disease, which is far less than the rate for patients diagnosed with localized stage disease (57%) [2]. Obviously, early detection contributes to favorable prognosis, and thus, early screening and diagnosis of cancer is of great significance.

Cancer screening tests have been used to detect different types of cancers at an early stage. The National Lung Screening Trial (NLST) showed that low‐dose helical computed tomography (CT) screening can reduce lung cancer mortality [4, 5]. However, not all of the cancers detected by screening with low‐dose CT will be found early. Furthermore, although low‐dose CT is a diagnostic method with high sensitivity, it often detects things that turn out not to be cancer [6]. Further follow‐up or invasive tests are required after screening for accurate diagnosis. As the advance of high‐throughput technologies, such as next‐generation sequencing, epigenetic alterations [7, 8, 9] of oncogenes or tumor suppressor genes have been investigated and emerged as the potential biomarkers for early diagnosis of cancers.

DNA methylation is a chemical modification of DNA by which methyl groups are added to the cytosines [10, 11]. Hypermethylation of tumor suppressor genes is a common event in various tumors, suggesting that DNA methylation alterations could be a new strategy for cancer diagnosis [12]. Compared with protein and genetic markers, DNA methylation signatures have a number of advantages. Methylation markers are relatively sensitive and stable than protein markers [12]. DNA methylation alterations often occur in the early stage of the cancer [13]. Moreover, methylation signatures can be detected in both cancer tissue and circulating tumor DNA which can be obtained in a minimally invasive manner [14]. In recent years, several candidate methylation markers have been studied in various cancer types [15, 16, 17], but none has been used in clinical practice yet. Integrative analysis of genome‐wide DNA methylation and gene expression has become an alternative method for systematically understanding the role of methylation variation in cancers with the potential of discovering new epigenetic markers that are more sensitive and robust.

In this study, we performed an integrative analysis of genome‐wide DNA methylation and gene expression data to identify DNA methylation markers for early diagnosis of LUAD. For this purpose, we used two public databases: The Cancer Genome Atlas (TCGA) and Gene Expression Omnibus (GEO). Using a machine learning approach, we finally identified three LUAD methylation markers including cg08032924, cg14823851, and cg19161124, mapped to *CMTM2*, *TBX4*, and *DPP6*, respectively. A logistic regression model based on the combination of these markers can accurately distinguish LUAD from normal samples on independent validation sets. The protein expression patterns of the markers were further validated by immunohistochemistry, and the suppression of tumor growth of *CMTM2* was confirmed in the mouse model.

## 2 Methods

### 2.1 DNA methylation

Illumina HumanMethylation450K array data of 415 LUAD and 31 associated normal tissues were downloaded from UCSC Xena (cohort: GDC TCGA Lung Adenocarcinoma) [18]. CpGs were annotated using human reference genome version 19 using IlluminaHumanMethylation450kanno.ilmn12.hg19 R package [19], and CpGs contain SNPs were removed from the analysis. Among the original data, the methylation profiles of paired adjacent normal tissues were available for 29 LUAD samples. Thus, these 29 primary tumors and matched adjacent normal samples were selected for differential methylation analysis. CpGs have missing values in less than 20% of the samples were imputed using mean methylation levels while those with more than 20% missing values were removed for differential analysis. The differentially methylated cytosines (DMCs) were reported with false discovery rate (FDR) < 0.05 (the Wilcoxon rank‐sum test) and methylation difference > 0.2. In addition, three independent validation datasets including GSE114989, GSE83842, and GSE85845 were obtained from the GEO [20]. GSE114989 [21] included 27 primary tumors and 7 matched normal tissues from 7 LUAD patients, GSE83842 [22] contained 12 cases with paired tumor and normal tissue, and GSE85845 [23] included 8 LUAD and adjacent nontumor tissues.

### 2.2 Gene expression

The HTSeq counts of RNA‐seq data for LUAD including 524 tumors and 59 normal samples were obtained from UCSC Xena, and the log‐transformed counts were converted into raw counts. Of the data, 18 primary tumors and matched adjacent normal samples were selected for differential expression analysis. The raw counts were normalized using the trimmed mean of *M* values (TMM) method, and edger [24] was used to perform the differential analysis. Differentially expressed genes (DEGs) were determined with adjusted *P*‐value < 0.05 and the log fold change > 1.5.

### 2.3 Clinical characteristics

The well‐preprocessed clinical information of the LUAD patients was obtained from [25] including basic characteristics such as sex, age at diagnosis, tumor stage, smoking status, and mutation status of genes such as *STK11*, *KRAS*, *KEAP1*, and *EGFR*. The survival data of the patients were obtained from UCSC Xena, and the Kaplan–Meier analysis with log‐rank test was used to compare overall survival across different groups.

### 2.4 Unsupervised clustering analysis

*K*‐means clustering algorithm with the Euclidean distance was used to determine the methylation subtypes of LUAD. Of the 54 429 promoter CpGs, 35 414 CpGs that were methylated (β > 0.05) in 32 normal tissues were removed, 18 859 CpGs showing low variations (σ < 0.2) in 153 tumor samples were removed, and finally 156 most variable CpGs were retained for clustering analysis.

### 2.5 Statistical analysis

The associations of methylation subtypes with clinical characteristics including sex, age at diagnosis, smoking history, tumor stage, smoking history, *STK11* mutation, *KEAP1* mutation, *KRAS* mutation, and *EGFR* mutation were examined using Fisher's exact test. The Kruskal–Wallis test was used to assess the statistical significance of differences in mean methylation levels among clusters. Pearson's correlation analysis was performed to assess the relationship between methylation status of CpGs and expression levels of genes.

### 2.6 Functional annotation

Gene ontology analysis was performed using the DAVID functional annotation tool [26], and significantly enriched (FDR < 0.05) biological processes and molecular functions were reported.

### 2.7 Marker identification by feature selection

Information gain [27], gain ratio [28], symmetrical uncertainty [29], and reliefF [30] in weka software package [31] were used for initial screening of the methylation markers. Information gain, gain ratio, and symmetrical uncertainty were all entropy‐based impurity measures. ReliefF considers differences in nearest neighbors to obtain the feature weights. All feature selection methods generate a score for each CpG that can be used to rank features. Top 15 scoring CpGs by each method were recorded. Default parameters were used for all methods except the number of selected attributes.

### 2.8 Immunohistochemical staining

Tissue microarrays (HLugA060PG02 and HLugA150CS03) were purchased from Shanghai Outdo Biotech Co., Ltd. (Shanghai, China), containing 105 human LUAD and paired normal adjacent lung tissues. The paraffin sections were stained with anti‐*TBX4* antibody (#sc‐515196; Santa Cruz Biotechnology, Shanghai, China) at a dilution of 1/10, anti‐*CMTM2* antibody (#PA5‐50208; Thermo Fisher Scientific, Shanghai, China) at a dilution of 1/150, and anti‐*DPP6* antibody (#sc‐365147; Santa Cruz Biotechnology) at a dilution of 1/50. For microwave antigen retrieval, Tris‐EDTA Buffer (pH 8.0) was employed, and multiple antigen retrievals were used if necessary. The *H*‐score was used for quantifying the protein levels in human LUAD and paired adjacent normal lung tissues. *H*‐score, ranging from 0 to 300, is the sum over product, which is calculated by multiplying the percentage of positive cells at each intensity and its staining intensity (weak, moderate, and strong were scored as 1, 2, and 3 based on color density).

### 2.9 Establishment of stable cell lines

To construct pLV‐EF1α‐Cmtm2‐IRES‐Bsd, DNA sequences encoding murine Cmtm2 were amplified from cDNA extracted from murine testis, then were cloned into the plasmid pLV‐EF1α‐MCS‐IRES‐Bsd (Biosettia, San Diego, CA, USA). Next, the lentiviruses carrying pLV‐EF1α‐Cmtm2‐IRES‐Bsd or pLV‐EF1α‐MCS‐IRES‐Bsd were packaged combined with commercial transfection agents, Lipofectamine 2000 (#11668027; Thermo Fisher Scientific). Mouse Lewis lung carcinoma (LLC) cells were incubated with the lentivirus‐containing supernatant with the presence of 8 μg·mL^−1^ polybrene for 48 h and followed by a selection with 10 μg·mL^−1^ blasticidin for one week to establish stable cell lines. The primers for amplification are mCmtm2 Forward 5′‐TCAACGCGTGCCACCATGGCAGCACCGATAAAGTTTCC‐3′ and mCmtm2 Reverse 5′‐TCAGCTAGCTTACCACTTCCTTAACCTA‐3′.

### 2.10 Real‐time quantitative PCR

Total RNA was extracted using TRIzol reagent (#15596026; Thermo Fisher Scientific) and then was under reverse transcription via the TransScript First‐Strand cDNA Synthesis SuperMix Kit (TransGen Biotech, Beijing, China). Quantitative PCR was performed on Roche real‐time PCR detection system using the following primers: Q‐Cmtm2 Forward 5′‐CCCAAAAAGGGGGCTTCGAC‐3′, Q‐Cmtm2 Reverse 5′‐ACCGGATGTGGGAGCATTGT‐3′.

### 2.11 Mouse model

Seven‐week‐old C57BL/6J mice (Vital River Laboratory Animal Technology Co. Ltd) were used and maintained in a specific pathogen‐free facility. Luciferase‐expressing Lewis lung carcinoma cells (1.5 × 10^5^) were injected subcutaneously into the right flank of C57BL/6J mice, and then, the volume of tumors was monitored every 3 days. And tumor weights were measured when the mice were sacrificed 4 weeks postimplant.

### 2.12 Bioluminescence imaging

For live imaging, the C57BL/6J mice were given intraperitoneal injections of the reporter substrate (15 mg·mL^−1^ stock in PBS, 100 μg·g^−1^ mouse) 10 min before imaging and then were transferred to the imaging chamber for imaging after anesthesia. Images were analyzed using Living Image software, and the fluorescence intensity was quantified.

### 2.13 Ethical approval

All the experiments involving mice were conducted according to the guidelines established by the Nankai University Animal Care and Use Committee (NUACUC) by skilled experimenters under an approved protocol, which was in accordance with the principles and procedures outlined in the NIH Guide for the Care and Use of Laboratory Animals.

## 3 Results

### 3.1 Global DNA methylation pattern in LUAD

**Fig. 1:**
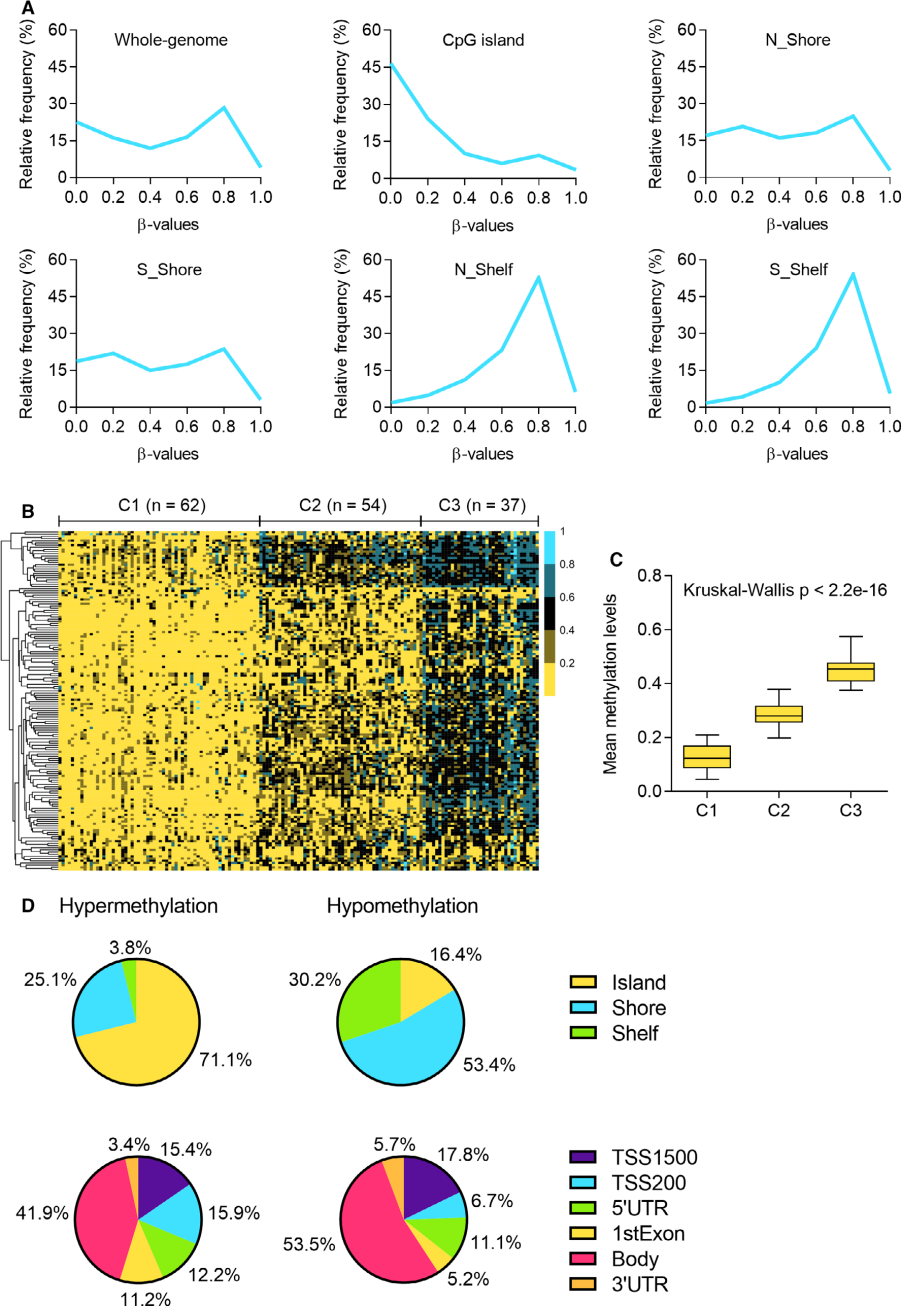
Genome‐wide DNA methylation patterns in LUAD. (A) The distribution of mean methylation levels of CpGs across 415 LUAD patients in whole‐genome CpG islands, north shores, south shores, north shelves, and south shelves. (B) Consensus clustering of 156 CpGs that varied most across 153 well‐annotated LUAD samples. Samples are presented in columns, and the CpGs are presented in rows. The methylation profile was clustered into three groups denoted as C1 (*n* = 62), C2 (*n* = 54), and C3 (*n* = 37). (C) The distributions of methylation levels in three clusters. Kruskal–Wallis test. (D) The distribution of hypermethylated and hypomethylated CpGs in different genomic regions including CpG island, shore, shelf, TSS1500, TSS200, 5′UTR, 1stExon, body, and 3′UTR.

To examine the global DNA methylation patterns in LUAD patients, the mean β value was calculated for each CpG dinucleotide across 415 tumors, and the distribution of methylation levels was examined in CpG islands (CGIs), shores, and shelves (Fig. 1A). A bimodal distribution was observed for all CpGs while a large hypomethylation (the peak in the left) was found for CpGs located in CGIs. In addition, the CpGs in both north and south shores had variable methylation levels (bimodal distribution) and the CpGs in north and south shelves had large hypermethylations (the peak in the right), indicating the CpGs within or near the CGIs tend to have low methylation levels, which is consistent with previous findings [32, 33]. We then performed an unsupervised clustering analysis of 156 CpGs that varied most across 153 well‐annotated LUAD samples (Fig. 1B). The DNA methylation profile of tumors was clustered into three distinct subtypes, which denoted C1 (*n* = 62), C2 (*n* = 54), and C3 (*n* = 37), and the mean beta values indicated a significant difference (Kruskal–Wallis *P* < 2.2e‐16) among clusters (Fig. 1C). Then, we investigated the association between the clusters and clinical characteristics, and sex, tumor stage, smoking history, *STK11* mutation, and *KEAP1* mutation were significantly (*P*‐value < 0.05) associated with clusters (Table 1). Kaplan–Meier survival analysis was performed to estimate overall survival of each cluster, and there were no significant differences among different clusters (*P*‐value = 0.46). Overall, hypermethylation of CGI was observed in LUAD patients as with in other cancer types, and the samples can clearly be divided into three methylation groups. However, high methylation was unlikely to be associated with poor survival.

**Table 1:** Clinical characteristics.

Characteristics	Classes	C1	C2	C3	*P*‐value
Sex	Female	34	37	15	0.030
	Male	28	17	22	
Age at diagnosis^a^	≥ 66	34	27	18	0.820
	<66	28	27	19	
Stage	Low (Stage I, Stage II)	44	47	25	0.044
	High (Stage III, Stage IV)	18	7	12	
Smoking history	Current or past smoker	59	40	34	0.003
	Lifelong nonsmoker	3	14	3	
*STK11* mutation	Mutant	18	7	4	0.038
	WT	44	47	33	
*KRAS* mutation	Mutant	22	16	14	0.691
	WT	40	38	23	
*KEAP1* mutation	Mutant	18	5	4	0.014
	WT	44	49	33	
*EGFR* mutation	Mutant	10	7	4	0.832
	WT	52	47	33	

^a^ Average age of the patients is 66.

Next, we analyzed the methylation differences in 29 LUAD and 29 matched normal samples. A total of 11 266 DMCs mapped to 3119 genes were detected, including 7415 hypermethylation (1687 genes) and 3851 hypomethylation (1432 genes) in tumors. We then investigated the distribution of hypermethylated and hypomethylated CpGs and genes in various genomic regions (Fig. 1D). Among hypermethylated CpGs, 71.1% were located in CGIs, 25.1% were in shores, and 3.8% were in shelves. However, 53.4% of hypermethylated CpGs were located in shores, 30% were in shelves, and only 16.4% were in CGIs. The variation of distribution between hypermethylated CpGs and hypomethylated ones was relatively smaller in gene‐context regions than in CGI‐based regions. Of hypermethylated (hypomethylated) CpGs, 15.4% (17.8%), 15.9% (6.7%), 12.2% (11.1%), 11.2% (5.2%), 41.9% (53.5%), and 3.4% (5.7%) were located in 1500‐bp upstream of transcription start site (TSS1500), 200‐bp upstream of TSS (TSS200), 5′ untranslated region (5′UTR), the first exon (1stExon), gene body, and 3′ untranslated region (3′UTR), respectively. It is obvious that the number of hypermethylated sites was higher in the regions near TSS. By differential methylation analysis, we identified 11 266 sites showing significant DNA methylation changes in tumors, and those DMCs were further used to be correlated with gene expression in downstream analysis to filter the DMCs that do not contribute to transcriptional regulation of genes.

### 3.2 Identification of relevant DNA methylation changes associated with mRNA expression

**Fig. 2:**
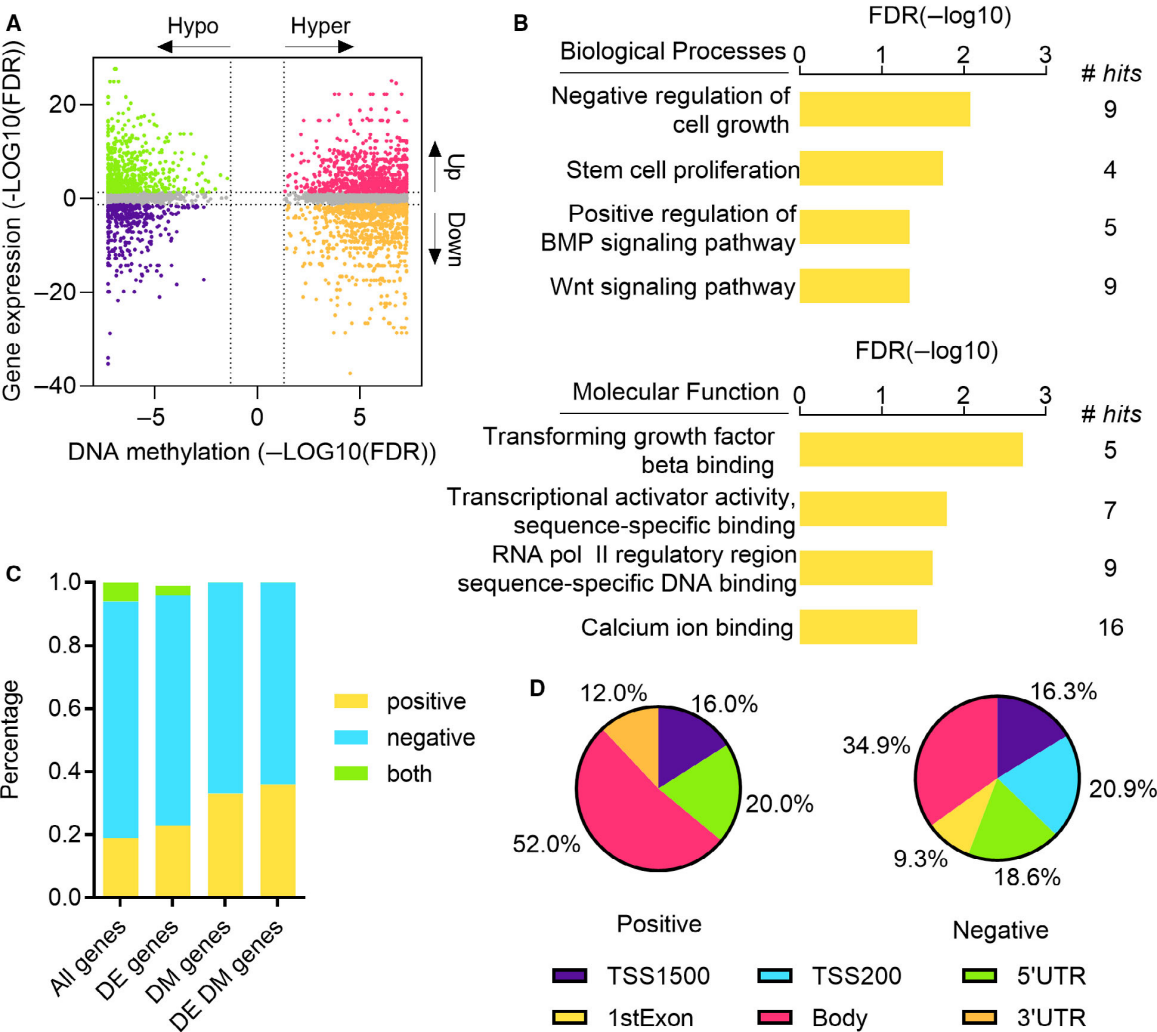
Joint analysis of DNA methylation and mRNA expression. (A) Starburst plot integrating DNA methylation changes and gene expression changes (*n* = 4297). The genes are divided into four groups that are hypermethylated and upregulated (pink); hypermethylated and downregulated (orange); hypomethylated and upregulated (green); hypomethylated and downregulated in LUAD (blue). (B) GO analysis for hypermethylated and downregulated genes. (C) Percentage of positive/negative correlation between DNA methylation and gene expression. Pearson's correlation coefficient was calculated for all genes, DEGs, differentially methylated genes, and differentially expressed and methylated genes. (D) The distribution of positive and negative correlations in different genomic areas.

We performed an integrated analysis of DNA methylation and gene expression to identify potentially relevant DNA methylation alterations in LUAD. Of the 29 matched tumor and normal samples for differential methylation analysis, 18 pairs that have expression profiles were used for differential expression analysis. A total of 2622 DEGs were detected including 1500 upregulated genes and 1086 downregulated genes. Of these genes, approximately one fifth of them showed significant methylation changes between LUAD and normal samples, including 134 (487 CpGs) hypermethylated and upregulated genes, 147 (383 CpGs) hypermethylated and downregulated genes, 128 (211 CpGs) hypomethylated and upregulated genes, and 82 (160 CpGs) hypomethylated and downregulated genes (Fig. 2A). Gene Ontology (GO) analysis was then performed to examine the biological functions of the 147 hypermethylated and downregulated genes (Fig. 2B). In biological processes, negative regulation of cell growth, stem cell proliferation, positive regulation of *BMP* signaling pathway, and *Wnt* signaling pathway were significantly enriched. In terms of molecular function, the genes were related to transforming growth factor beta binding, transcriptional activator activity, sequence‐specific binding, RNA polymerase II regulatory region sequence‐specific DNA binding, and calcium ion binding. Cancer‐related pathways such as *Wnt* signaling pathway were enriched in hypermethylated and downregulated groups.

Next, we performed a correlation analysis to assess the relationship between DNA methylation and gene expression. Pearson's correlation coefficients were calculated between 281 938 CpGs and corresponding genes (Fig. 2C). Using a coefficient cutoff of 0.3, 595 genes (19%) were positively correlated with methylation while 2409 genes (75%) were negatively correlated with methylation. Similar patterns were observed when considering correlations in DEGs, differentially methylated genes, and differentially expressed and methylated genes. Note that multiple CpGs can be associated with a gene, and the methylation of those CpGs can be both positively and negatively correlated with gene expression. Then, we examined the distribution of CpGs that are significantly correlated with genes in different genomic regions (Fig. 2D). Of the CpGs positively affected gene expression, approximately half of them were located in gene body, 20% were in 5′UTR, 16% were in TSS1500, and 12% were in 3′UTR. Of the CpGs negatively regulated gene expression, 34.9% were located in gene body, 20.9% were in TSS200, 18.6% were in 5′UTR, 16.3% were in TSS1500, and 9.3% were in 1stExon. Interestingly, all CpGs in TSS200 and 1stExon were negatively correlated with gene expression. These results reveal that the CpGs located near TSS tend to negatively regulate expression of genes while the CpGs in gene body tend to positively regulate gene expression.

### 3.3 Identification and validation of methylation signatures in LUAD

**Fig. 3:**
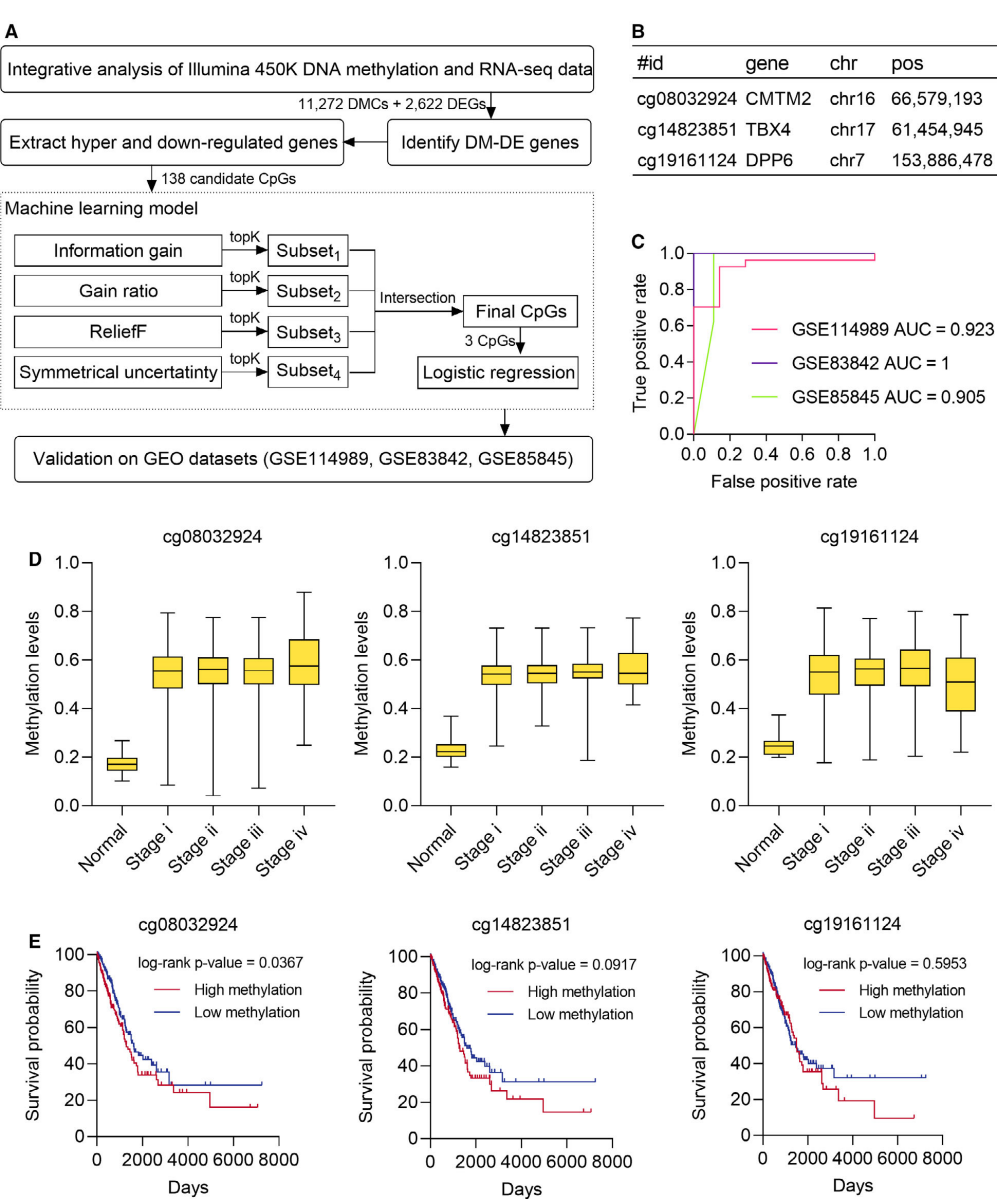
Identification of LUAD methylation markers. (A) The framework of identifying LUAD methylation markers. (B) The genomic details of discovered three methylation markers. (C) Receiver operating characteristic (ROC) curves and AUC values on validation sets. (D) The distribution of methylation levels of three markers in different tumor stages (*n* = 495). (E) The Kaplan–Meier survival curves for three methylation markers. The boundary for high and low methylation was the average methylation level.

To identify methylation markers for LUAD diagnosis, the candidate markers were further narrowed down to hypermethylated and downregulated CpGs since the repression of tumor suppressor genes by the promoter hypermethylation is one of the most frequently observed epigenetic alterations in cancers. Of the 383 hypermethylated and downregulated CpGs, we selected 138 CpGs in promoter regions as a candidate functionally relevant group, and machine learning techniques were adopted to determine the final methylation markers in LUAD (Fig. 3A). We used four different feature selection approaches that were information gain (IG), gain ratio (GR), symmetrical uncertainty (SU), and reliefF (RF) to screen the markers. We extracted top 15 ranked CpGs found by each method and took the intersections of those CpGs as the final methylation markers (Table 2). Finally, we identified three methylation markers that were cg08032924, cg14823851, and cg19161124, mapped to *CMTM2*, *TBX4*, and *DPP6*, respectively (Fig. 3B). A logistic regression model was then built with these markers on TCGA LUAD samples, and the model was validated on three independent datasets from GEO. The areas under the receiver operating characteristic curve (AUCs) were 0.923, 1, and 0.905 for GSE114989, GSE83842, and GSE85845,, respectively, indicating that the three markers can accurately classify LUAD samples from controls (Fig. 3C). To examine whether these markers were suitable for early detection of LUAD, we compared the methylation levels of TCGA LUAD patients in different tumor stages. All the markers were found to be significantly (Mann–Whitney *P*‐value < 0.0001) hypermethylated in stage I tumors compared to normal samples (Fig. 3D). In addition, the patients were divided into high methylation and low methylation groups based on the average methylation levels of the markers, and the Kaplan–Meier analysis was conducted to investigate the association between the methylation status of the markers and the overall survival of patients (Fig. 3E). For cg08032924 and cg14823851, patients with low methylation levels had significantly better survival than those with high methylation levels (*P* = 0.0367 and *P* = 0.0917). For cg19161124, the overall survival between the two groups was not statistically significant (*P* = 0.5953) but the low methylation group still had better survival than the high methylation group. These results suggest that the identified markers can accurately predict LUAD and also work well on early‐stage patients.

**Table 2:** Top 15 ranked CpGs (genes) identified by different feature selection methods for diagnosis of LUAD.

IG	GR	SU	RF
***cg14823851:TBX4***	***cg14823851:TBX4***	***cg14823851:TBX4***	***cg14823851:TBX4***
*cg01158277:CRYAB*	*cg09523275:NKAPL*	*cg10253847:NKAPL*	*cg22620221:DPP6*
*cg10253847:NKAPL*	*cg18252309:DPP6*	*cg18252309:DPP6*	***cg19161124:DPP6***
*cg18252309:DPP6*	*cg10253847:NKAPL*	*cg18694169:NKAPL*	*cg25075794:AQP1*
*cg18694169:NKAPL*	*cg18694169:NKAPL*	*cg09523275:NKAPL*	*cg04372674:AQP1*
*cg09523275:NKAPL*	***cg19161124:DPP6***	***cg19161124:DPP6***	*cg01031101:NKAPL*
***cg19161124:DPP6***	*cg19797376:TAL1*	*cg07153665:CMTM2*	***cg08032924:CMTM2***
*cg07153665:CMTM2*	*cg07153665:CMTM2*	***cg08032924:CMTM2***	*cg18674980:CA3*
*cg06499647:C2orf40*	*cg21838979:C2orf40*	*cg19797376:TAL1*	*cg14535980:C2orf40*
*cg21838979:C2orf40*	***cg08032924:CMTM2***	*cg21838979:C2orf40*	*cg25230363:AQP1*
*cg19797376:TAL1*	*cg06499647:C2orf40*	*cg06499647:C2orf40*	*cg10402698:SMAD6*
*cg09854734:CMTM2*	*cg05546863:CMTM2*	*cg09854734:CMTM2*	*cg19908768:SULT1C4*
***cg08032924:CMTM2***	*cg16626067:CMTM2*	*cg05546863:CMTM2*	*cg04567731:TBX4*
*cg14535980:C2orf40*	*cg09854734:CMTM2*	*cg14535980:C2orf40*	*cg07510423:C2orf40*
*cg17384889:NKAPL*	*cg17384889:NKAPL*	*cg16626067:CMTM2*	*cg01158277:CRYAB*

The markers commonly identified by four methods (in bold).

### 3.4 *CMTM2* and *TBX4* are weakly expressed in human LUAD

**Fig. 4:**
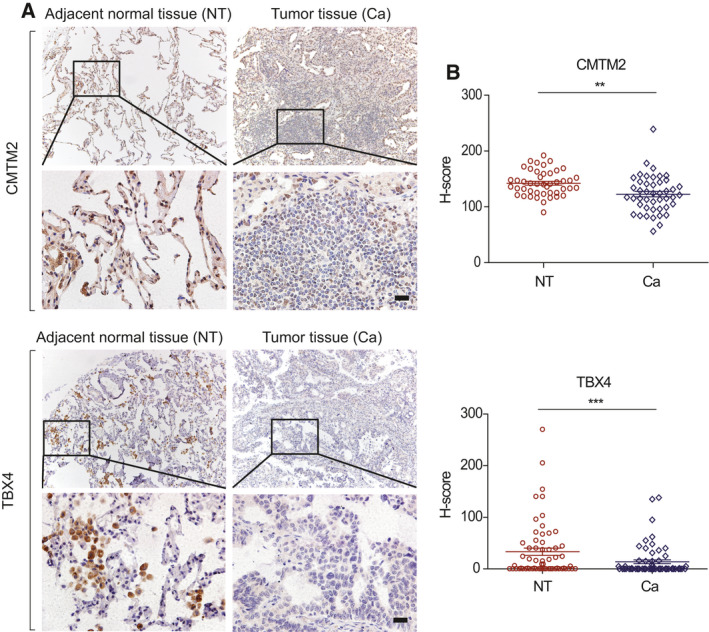
*CMTM2* and *TBX4* are weakly expressed in human LUAD. (A) Representative IHC images of tissue arrays containing human LUAD specimens and paired adjacent normal tissues. Regions in squares are magnified 4× in bottom panels. Scale bar represents 20 μm. (B) Summary statistics of *H*‐score based on only intact and paired specimens, *n* = 45 for *CMTM2* and *n* = 61 for *TBX4*. Student's *t*‐test. **P‐value < 0.01, ***P‐value < 0.001.

To further confirm the correlation between the three newly identified hypermethylated genes and LUAD progression, immunohistochemistry (IHC) was performed to evaluate the protein expression of *CMTM2*, *TBX4,* and *DPP6* in tissue arrays containing 105 human LUAD specimens and paired adjacent normal tissues. Quantitative analysis based on intact and paired specimens via *H*‐score revealed that the expression of *CMTM2* and *TBX4* was obviously lower in LUAD when compared to the paired adjacent normal lung tissues, implying the potential gene silencing of *CMTM2* and *TBX4* caused by DNA methylation in LUAD (Fig. 4), whereas the correlation between *DDP6* and LUAD remains to be further investigated since *DPP6* might have low expression even in the normal lung cells (Fig. S1). The protein expression patterns of *CMTM2* and *TBX4* in LUAD were consistent with their hypermethylation profiles previously identified, confirming that cg08032924 and cg14823851, mapped to *CMTM2* and *TBX4*, are potential novel methylation markers in human LUAD.

### 3.5 *CMTM2* decreases tumor growth of mouse Lewis lung carcinoma *in vivo*

**Fig. 5:**
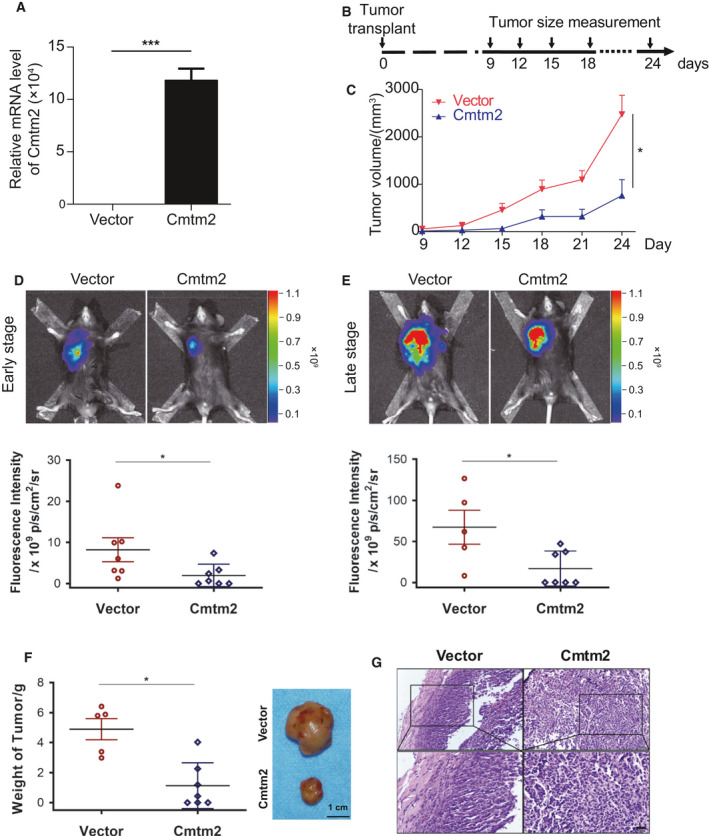
*Cmtm2* decreases tumor growth *in vivo*. (A) The mRNA level of *Cmtm2* is overexpressed in LLC cells (*n* = 3). Student's *t*‐test. ***P‐value < 0.001. (B) Schematic of mouse model on C57BL/6J mice (*n* = 14). (C) Tumor volume of LLC‐bearing mice (*n* = 14). Student's *t*‐test. *P‐value < 0.05. (D, E) Representative images and quantification of bioluminescence imaging in early (D) (*n* = 14) and late stage (E) (*n* = 12, 2 died) post‐tumor implant. (F) Weight and representative images of excised tumors (*n* = 14). (G) Representative images of HE staining of tumors. Scale bar represents 20 μm. Student's *t*‐test. *P‐value < 0.05.

Considering potential clinical significance and biologic implications, we focused on *CMTM2* for further research as the immunohistochemical analysis demonstrates that *CMTM2* is extensively expressed in human lung tissues in our study, implying its comprehensive significance. To elucidate the role of *Cmtm2* in LUAD, we examined the effects of *Cmtm2* on mouse Lewis lung carcinoma (LLC) *in vivo*. The mRNA level of *Cmtm2* revealed that the *Cmtm2* was successfully ectopically expressed in LLC cells (Fig. 5A). Subcutaneously implanted tumor model was applied to C57BL/6J mice to investigate the effects of *Cmtm2 in vivo* (Fig. 5B). Meanwhile, the tumor growth was monitored by measuring the tumor volume (Fig. 5C) as well as living imaging (Fig. 5D,E). It is noticeable that the elevation of *Cmtm2* significantly suppressed the subcutaneous tumor growth compared with the control group (Fig. 5C–F) even in the early stage (Fig. 5D). As expected, the weight of tumors was diminished by *Cmtm2* robustly when the mice were sacrificed after 4 weeks (Fig. 5F,G). These results suggest that *Cmtm2* could suppress the tumor growth of LUAD *in vivo*.

## 4 Discussion

In this study, we explored the global DNA methylation patterns in LUAD and identified three subgroups showing distinct methylation status. CpG island methylator phenotype (CIMP) is characterized by strong hypermethylation of CpG islands in the promoter regions of tumor suppressor genes, and previous studies have reported that CIMP is associated with patient outcomes in various cancers including colorectal cancer, hepatocellular carcinoma, and gastric cancer [34, 35]. The association between CIMP high group and overall survival of LUAD patients remains unclear since there are some discrepancies among different genome‐wide methylation studies. Karlsson *et al*. [36] have reported that CIMP shows differences in adenocarcinomas and it is associated with mutation frequency of common tumor suppressor genes such as *KEAP1, TP53, STK11, and SMARCA4*. However, Vaissière *et al*. [33] and Selamat *et al*. [37] have shown that CIMP is unlikely to be present in LUAD. Our analysis results demonstrated that DNA methylation subgroups were associated with genetic and clinical characteristics including sex, stage, smoking history, *KEAP1*, and *STK11* mutation but there is no evidence for poorer overall survival in CIMP group.

Functional annotation analysis revealed that hypermethylated and downregulated genes were enriched in cancer‐related pathways such as *Wnt* signaling pathway. A number of researches have shown that *Wnt* signaling pathway is important in the development of lung cancer. Mazieres *et al*. [38] have reported that aberrant methylation of *Wnt* inhibitory factor‐1 (*WIF‐1*) is an important cause of constitutive activation of the *Wnt* pathway in lung cancer. Selamat *et al*. [37] have shown that sclerostin domain containing 1 (*SOSTDC1*), a secreted regulator of *Wnt* pathway, is hypermethylated and downregulated in LUAD. Consistent with these results, hypermethylation of *WIF‐1* and *SOSTDC1* was observed in our study and the mRNA levels of these two genes were decreased in LUAD. Additionally, hypermethylation of other *Wnt* inhibitors including *RSPO1, RSPO2, RSPO4, WNT3A, DKK2, NKD1*, and *TMEM88* was also observed in our study.

To further screen reliable methylation markers, we designed a machine learning framework and identified discriminative CpGs for accurately distinguishing LUAD from normal samples. Feature selection is a machine learning technique, which determines the most relevant features for the target problem [39]. Feature selection has been widely used in biological and medical applications including gene expression analysis [40], transcription factor binding motif analysis [41], and drug discovery [42]. Here, we considered the marker screening as a problem of selecting a relevant feature (CpG) subset for predicting LUAD. Thus, four feature selection models were selected as the initial screening and the results of them were combined together by taking the intersections. Finally, we identified three methylation markers that were cg08032924 (*CMTM2*), cg14823851 (*TBX4*), and cg19161124 (*DPP6*), and the logistic regression model trained using these markers can accurately predict LUAD in independent validation datasets from GEO. *CMTM2* (CKLF Like MARVEL Transmembrane Domain Containing 2) is a protein coding gene that belongs to the chemokine‐like factor gene superfamily. *CMTM2* may play an important role in testicular development and is highly expressed in normal adult human testis in a stage‐specific manner. Lower expression of *CMTM2* is correlated with spermatogenesis defects including spermatogenesis arrest, which indicates that *CMTM2* might be involved in spermatogenesis [43]. *CMTM2* was found to be expressed at significantly lower level in Sézary syndrome (Sz), an aggressive type of cutaneous T‐cell lymphoma, than in benign T‐cell samples, and hypermethylation of *CMTM2* promoter can distinguish Sz from erythroderma secondary to inflammatory skin diseases [44]. *TBX4* is a transcription factor that belongs to a phylogenetically conserved family of genes that share a common DNA‐binding domain, the T‐box. *TBX4* was found to be expressed in hindlimb, lung, and proctodeum, and it plays an important role in the development of the hindlimb and in the formation of the umbilicus. A study of a total of 119 bladder cancer samples analyzed by Infinium methylation array showed that *TBX4* was differentially methylated in bladder cancer and was related to disease progression [45]. The methylation of *TBX4* promoter has not been reported to be associated with LUAD, though it has been observed to be downregulated in human non‐small‐cell lung cancer (NSCLC). Lai *et al*. [46] investigated the expression of a long noncoding RNA *TTTY15* in 37 NSCLC samples and found that downregulation of *TTTY15* was associated with poor prognosis. Interestingly, they also reported that *TTTY15* positively regulated *TBX4* expression by interacting with DNMT3A to affect the binding ability of DNMT3A to the *TBX4* promoter. *DPP6* is a single transmembrane protein that belongs to the peptidase S9B family of serine proteases and is most known for promoting cell surface expression of the potassium channel KCND2. Hypermethylation and decreased expression of *DPP6* were observed in endometrial cancer [47] and melanoma [48] while the role of *DPP6* in LUAD is still unclear.

Early detection, screening, and diagnosis of cancer greatly improve the patient survival rates, as well as significantly reduce the cost and increase the chances for successful treatment. Aberrant DNA methylation plays an important role in cancers and has shown to be a potential biomarker for the early detection of cancer. Compared with other biomarkers such as protein, methylation signature is relatively stable over time and involved in the early stage of carcinogenesis [49]. Moreover, DNA can be isolated with high quality and sufficient yield from frozen biospecimens [50], and DNA methylation status of various gene promoters can be easily captured from biological samples that can be obtained noninvasively including urine, blood, saliva [51]. Epi proColon is an FDA‐approved methylation assay that diagnoses colorectal cancer based on methylation status of the target DNA sequence in the promoter region of the *SEPT9*. Methylation markers have also been evaluated for early detection of prostate cancer, and a number of studies have shown that tissue‐based *GSTP1* methylation assay can achieve relatively high sensitivity (~ 80%) compared with prostate‐specific antigen testing [52, 53]. A number of researches have also been done to identify cancer‐specific DNA methylation markers in lung cancer patients. Yan *et al*. [54] identified a panel with nine CpGs using a combined public methylation datasets and constructed a prognosis model to predict survival in LUAD patients. Diaz‐Lagares *et al*. [13] identified four methylation markers including *BCAT1*, *CDO1*, *TRIM58*, and *ZNF177* and achieved 85% AUC on a regression model trained from the combination of four markers in bronchoalveolar lavages from patients with lung cancer. In our study, we identified three methylation markers and a logistic regression model trained with these markers on TCGA LUAD samples achieved high AUCs on three independent validation sets. Moreover, we observed these three markers were significantly hypermethylated in stage I LUAD patients, indicating these markers have a great potential to be used to detect LUAD at an early stage. Although the markers have high sensitivity, further validations on different populations are needed and the methylation status of there markers should be validated using cost‐effective technology, such as PCR‐based methods, in both LUAD tissues and noninvasive samples. In addition, the downstream functions of the marker genes will be systemically studied on our future work.

## 5 Conclusions

In summary, we integrated genome‐wide DNA methylation and mRNA expression data and identified three methylation signatures including cg08032924 (*CMTM2*), cg14823851 (*TBX4*), and cg19161124 (*DPP6*) for early diagnosis of LUAD. The results revealed that these markers can distinguish LUAD from normal samples with extremely high AUCs. The decreased expressions of *CMTM2* and *TBX4* were further confirmed in LUAD tissues by IHC. Moreover, we demonstrated that *Cmtm2* could suppress the tumor growth of LUAD *in vivo*. We believe that our study lays the foundation for further biological mechanisms of LUAD development and can contribute to the improvements in early detection and intervention for lung cancer.

## Conflict of interest

The authors declare no conflict of interest.

## Author contributions

LW, RX, YS, and YP were responsible for the conception and design of the study. ML and YP contributed to the data analysis. ML, CZ, SL, LZ, and LW conducted the biological validation. ML, YJC, LW, YS, and YP drafted and revised the manuscript. All authors revised, read, and approved the final manuscript.

## Code availability

The source codes are available at: https://sourceforge.net/projects/luad/files/

## Supporting information


**Fig. S1.** DPP6 is expressed in a low level.

## Data Availability

Illumina HumanMethylation450K array, RNA‐seq, and clinical information were available at UCSC Xena browser (cohort: GDC TCGA Lung Adenocarcinoma). Three independent validation sets were available at GEO database (accession: GSE114989, GSE83842, and GSE85845).
