# Supplementary material for: Identification and validation of novel DNA methylation markers for early diagnosis of lung adenocarcinoma
Source: Mol Oncol. 2020 Aug 27;14(11):2744–58. doi: 10.1002/1878-0261.12767 (PMC7607165; doi:10.1002/1878-0261.12767)
Supplement: Supplementary file 1 — Fig. S1. DPP6 is expressed in a low level. [file MOL2-14-2744-s001.docx]

Supplementary Materials for

**Identification and validation of novel DNA methylation markers for early diagnosis of lung adenocarcinoma**

Miao Li^1^, Chen Zhang^1^, Lijun Zhou^1^, Siyu Li^1^, Yuan Jie Cao^3^, Longlong Wang^1,2^, Rong Xiang^1^, Yi Shi^1,2,*^, Yongjun Piao^1,2,*^

^1^School of Medicine, Nankai University, Tianjin, China.

^2^Tianjin Key Laboratory of Human Development and Reproductive Regulation, Nankai University Affiliated Hospital of Obstetrics and Gynecology, Tianjin, China.

^3^Department of Radiation and Oncology, Tianjin Medical University Cancer Institute and Hospital, National Clinical Research Center for Cancer and Tianjin Key Laboratory of Cancer Prevention and Therapy, Tianjin, China

^*^Co-correspondence: Yongjun Piao (ypiao@nankai.edu.cn) and Yi Shi (yishi@nankai.edu.cn)


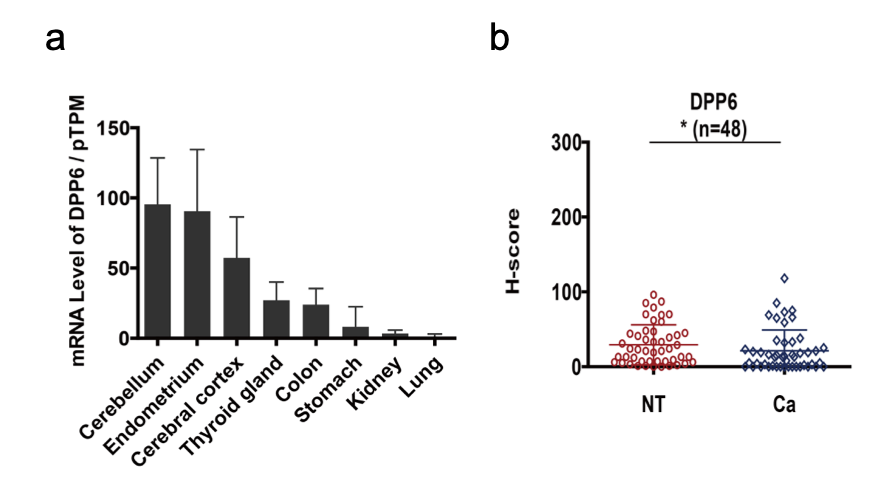


Fig S1. DPP6 is expressed in a low level. a. mRNA level of DPP6 in different tissues. b. DPP6 protein levels in cancer and normal adjacent tissues, n=48. Student's t test. *p-value < 0.05.
